# Shortness of breath after AV ablation: case of left phrenic nerve palsy

**DOI:** 10.3402/jchimp.v3i1.19123

**Published:** 2013-04-17

**Authors:** Irene Lambiris, Jinesh Mehta, Marcelo Helguera

**Affiliations:** Department of Cardiology/Electophysiology, Pulmonary Critical Care and Internal Medicine, Cleveland Clinic, Weston, FL, USA

**Keywords:** phrenic nerve palsy, atrial fibrillation, cardiac ablation, chronic obstructive pulmonary disease

## Abstract

Phrenic nerve palsy has been recognized as a complication of catheter ablation with a prevalence of 0.11–0.48% after atrial fibrillation ablation, independent of the type of ablation catheter or energy source, likely due to the anatomical relationship of the nerves. This report describes a case of new onset of shortness of breath (SOB) due to left diaphragm paralysis following transcatheter radiofrequency ablation in a patient with underlying chronic obstructive pulmonary disease.

A 58-year-old man with a 6-year history of paroxysmal atrial fibrillation (PAF), CHADS2=0, was referred to be evaluated for candidacy for catheter ablation. He had recurrent, symptomatic PAF despite treatment with atenolol. Initially, these episodes were sporadic and brief, but more recently were occurring 2–3 times per week. His last episode was the day before his appointment. The longest episode to date had been documented as 10 hours. These episodes were associated with palpitations, shortness of breath (SOB), fatigability, weakness, and general sense of ill being. The patient had no history of anti-arrhythmic use, and had never been cardioverted. He was on dabigatran for anti-coagulation. Outside the PAF, he felt well and was fully functional, running 3 miles daily. He had no other significant medical problems. His physical examination was unremarkable. He had no structural heart disease. Prior two-dimensional echocardiography revealed normal left ventricular ejection fraction of 50%. A holter monitor revealed a normal sinus rhythm, heart rate (HR) from 41 to 147 beats per min (bpm), average HR 56 bpm, nine premature ventricular contractions, and 40 atrial premature contractions. Electrocardiogram at time of evaluation showed sinus bradycardia at 45 bpm. The patient was not interested in chronic anti-arrhythmic drug treatment. He was seeking a potentially curative approach. The patient was a good candidate for catheter ablation consisting of pulmonary vein antral isolation and was scheduled for the next day after obtaining a trans esophageal echocardiogram (TEE) and computed tomography (CT) angiogram of the chest. TEE revealed no left atrial/left atrial appendage thrombus, and normal left ventricular size and function/ejection fraction (EF) of 55%. A CT angiogram demonstrated pulmonary venous anatomy in the spectrum of normal limits (Fig. 1).

**Fig. 1:**
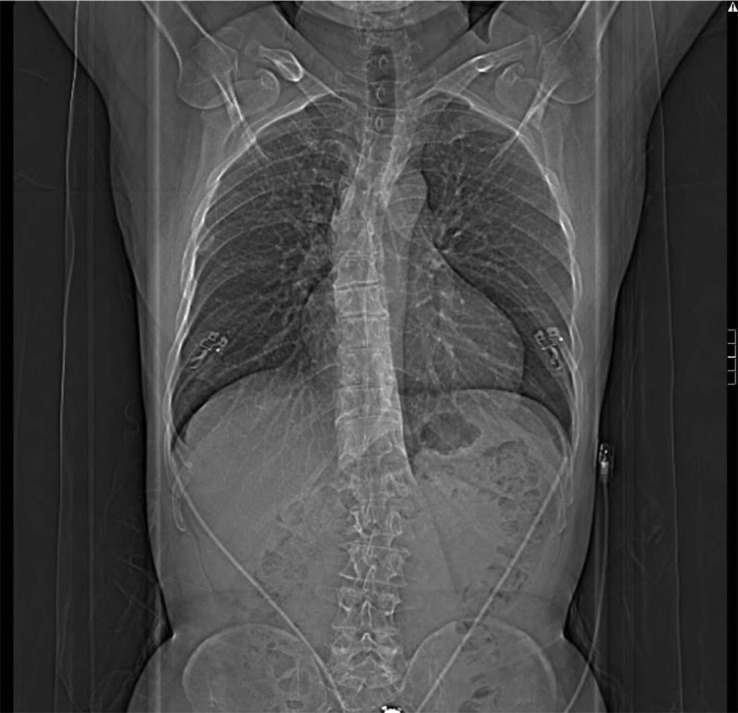
8/2011 Visualized lungs demonstrate mild centrilobular emphysema.

The team proceeded with double transseptal puncture. A contrast-enhanced CT was obtained in advance of the procedure and imported into the electroanatomic mapping system. During ablation of the posterior aspect of the pulmonary vein, the authors did not suspect left phrenic nerve injury. The patient recovered from anesthesia well. One month after the procedure, the patient returned for further evaluation of shortness of breath after running his usual 3 miles. The patient had noticed that his average 7-min mile has now been increased to a 12-min mile, and that his normal exercise capacity was limited due to dry heaves and shortness of breath. He denied cough, orthopnea, leg swelling, syncope, shortness of breath at rest, dizziness, palpitations, ill being, or chest pain. He stated that he had not had any episode of irregular heart rhythm. A pulmonary referral ensued and the patient was worked up for a centrilobar emphysema seen on a CT scan, with repeat radiological images, alpha 1 anti-trypsin levels, and PFTs. Results showed Alpha 1 AT-31 (normal), Chest X-Ray (Fig. 2): elevated left hemi-diaphragm. CT of the chest (Fig. 3): Mild centrilobular emphysema and new elevated left hemi-diaphragm. Pulmonary function tests (PFTs) findings were as follows: Reduced FEV1 consistent with mild obstruction and normal lung volumes. The patient was given a trial of an albuterol inhaler and a chest fluoroscopy (Fig. 3) was ordered to evaluate elevated left hemi-diaphragm. Images were consistent with left phrenic nerve palsy. Nine months after the AV ablation, the patient has been in sinus rhythm, not necessitating anti-arrhythmics, but still suffers from shortness of breath and limited exercise capacity. Repeat chest fluoroscopy in 4/2012 showed persistent left phrenic nerve paralysis.

**Fig. 2:**
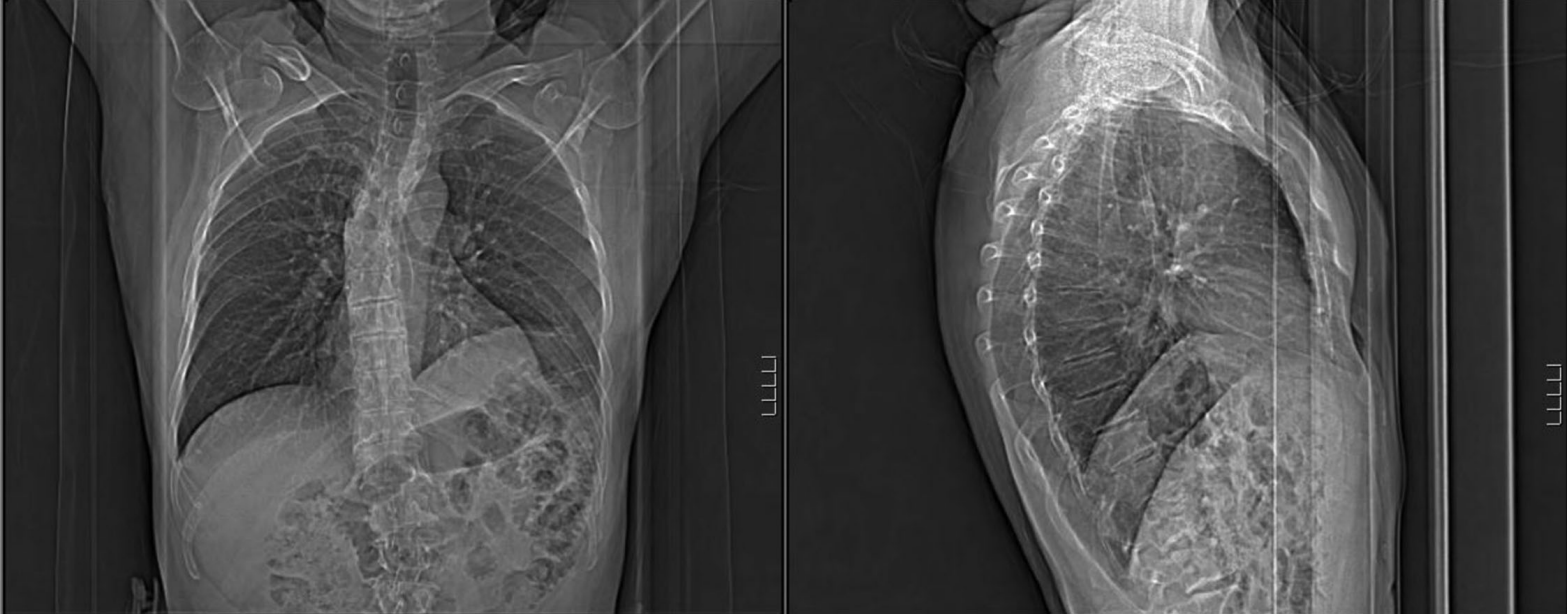
11/2011 Mild centrilobular emphysema. Elevated left hemi diaphragm.

**Fig. 3:**
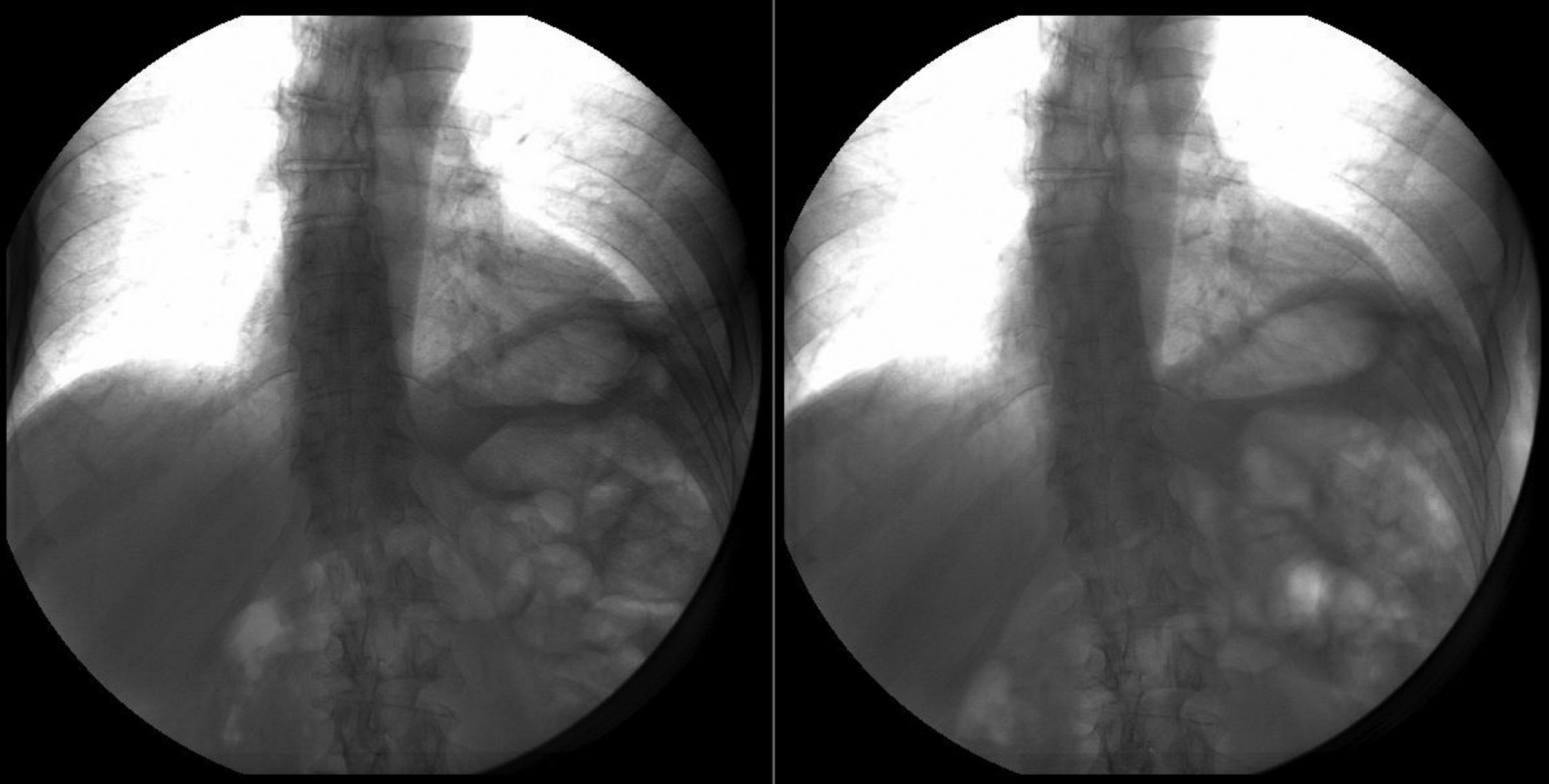
11/2011 Chest fluoroscopy/sniff test.

## Discussion

It is well known that the left phrenic nerve runs posterior to the subclavian vein and has a well-established anatomical proximity to the left atrial appendage (1). The right phrenic nerve passes laterally over the superior vena cava (SVC), right atrium, and the anterior–inferior part of the right superior pulmonary vein (PV). Therefore, caution is required when dealing with the infero-anterior part of the PV ostium, the postero-septal part of the SVC, and the proximal left atrial appendage roof (2).

Phrenic nerve injury is a rare, normally benign complication of a catheter ablation procedure (3,4). Anatomically the nerve is indeed in harm's way (5,6). The left phrenic nerve, for example, descends closely over the left atrial appendage and passes along the pericardium and left ventricle, in close relation to the epicardial cardiac veins in some individuals (7). The phrenic nerve is more sensitive to damage from heat than the myocardium because it tends to have insulator properties that retain heat (8). The radiofrequency delivery can alter the action potential duration and amplitude of the susceptible nerve, causing a transient conduction delay (7). The longer the duration of the exposure, the more extensive the damage. Diligent monitoring of phrenic nerve function during ablation through high output pacing can prevent accidental injury. Most published studies have documented that up to 50% of patients with phrenic nerve injury remain asymptomatic, dyspnea being the most common symptom in symtpomatic patients (9). The average effect of nerve paralysis due to AV ablation has typically been transitory. Eighty percent of patients have complete resolution between 6 and 28 months post-ablation. Our patient is 9 months post-procedure, with continued residual left diaphraghm paralysis proven by repeat flurosocopy, and SOB, limiting his exercise capacity. What is unique about our patient is, although he denies any smoking or pulmonary history, his baseline CT scan and PFTs indicate COPD/emphysema. Phrenic nerve injury (PNI) alone would give us a restrictive disease pattern on PFTs. One can surmise that his underlying pulmonary problem may be the cause of his delayed recovery, thus necessitating not only high output pacing prior to ablation, flurosocopy monitoring of diaphragmatic motility during ablation, and energy delivery away from Phrenic Nerve (PN), but also, more importantly, risk stratifying those patients who may be more susceptible to PNI, due to underlying lung disease (7).
